# Comparison of methods to estimate water‐equivalent diameter for calculation of patient dose

**DOI:** 10.1002/acm2.12383

**Published:** 2018-07-07

**Authors:** Andrew Daudelin, David Medich, Syed Yasir Andrabi, Chris Martel

**Affiliations:** ^1^ Worcester Polytechnic Institute Worcester MA USA; ^2^ Lahey Hospital & Medical Center Burlington MA USA; ^3^ Philips Healthcare Andover MA USA

**Keywords:** computed tomography, size‐specific dose estimate

## Abstract

Modern CT systems seek to evaluate patient‐specific dose by converting the CT dose index generated during a procedure to a size‐specific dose estimate using conversion factors that are related to patient attenuation properties. The most accurate way to measure patient attenuation is to evaluate a full‐field‐of‐view reconstruction of the whole scan length and calculating the true water‐equivalent diameter (*D*_w_) using CT numbers; however, due to time constraints, less accurate methods to estimate *D*_w_ using patient geometry measurements are used more widely. In this study we compared the accuracy of *D*_w_ values calculated from three different methods across 35 sample scans and compared them to the true *D*_w_. These three estimation methods were: measurement of patient lateral dimension from a pre‐scan localizer radiograph; measurement of the sum of anteroposterior and lateral dimensions from a reconstructed central slice; and using CT numbers from a central slice only. Using the localizer geometry method, 22 out of 35 (62%) samples estimated *D*_w_ within 20% of the true value. The middle slice attenuation and geometry methods gave estimations within the 20% margin for all 35 samples.

## 1 INTRODUCTION

The volumetric Computed Tomography Dose Index (CTDI_vol_) provided by CT scanners is a calculated quantity representing the dose delivered to a standardized, homogeneous calibration phantom of a specified size based on CT parameter settings used during the scan.^1^ Because the CTDI_vol_ does not account for an individual patient's size or attenuation properties, it therefore is not a direct measurement of the absorbed dose delivered to a patient.^2^ To address this, the size‐specific dose estimate (SSDE), which modifies CTDI_vol_ using a factor related to patient size, was introduced by the American Association of Physicists in Medicine (AAPM) in 2011.^3^ As part of this effort, the AAPM Task Group 204 developed size‐specific conversion factors (*k*) to better estimate patient radiation absorption properties and size‐specific doses. These conversion factors are multiplied by CTDI_vol_ to obtain the SSDE. Members of AAPM Task Group 220^4^ further developed the technique by using the attenuation of x rays through the body, as measured by the CT scanner, to calculate patient water‐equivalent diameter (*D*_w_), the diameter of a cylindrical volume of water with equivalent mean attenuation. *D*_w_ is a more precise metric of body size for the selection of a conversion factor because it accounts for radiation absorption directly by using attenuation information.^5^, ^6^

*D*_w_ may be calculated directly from a full‐field‐of‐view reconstruction,^7^ or estimated using the geometric measurement methods of TG204. Geometric estimation requires the use of additional corrections based on the body region scanned to account for differences in attenuation of abdominal and thoracic anatomy. Calculation of *D*_w_ using reconstructed attenuation values is more patient‐specific and uses data directly relevant to the metric of interest. It is therefore the preferred method for determining the appropriate conversion factor.^4^ The reconstructed region is ideally the full scan range, though Leng et al. showed that *D*_w_ calculation from a central slice can be an acceptable substitute.^8^ Anam et al. demonstrated that a fully automated image processing and *D*_w_ calculation method can match manual calculation across a range of scan regions in both phantoms and human patients.^9^

The purpose of this paper is to compare *D*_w_ values from three estimation methods to a reference standard *D*_w_ value calculated using a full‐field‐of‐view, full scan range reconstruction. The first method investigated was measurement of patient lateral dimensions (LAT) from a localizer radiograph image. The second method was measurement of patient lateral and anteroposterior (AP) dimensions and calculation using an equation from TG204. The final method was a calculation of *D*_w_ using attenuation values from a central slice only. All of these estimation methods are less time‐ and resource‐intensive than a full scan reconstruction, so hospital resources could be used more efficiently if any are found to be an acceptable substitute. Additionally, localizer radiograph images include the full scan range with no truncation, whereas reconstructions are often truncated to include only the region of interest, resulting in the loss of attenuation information from other irradiated anatomy.

## 2 MATERIALS AND METHODS

### 2.A Selection of image data sets

CT scan data from 35 sets of anonymized patient scan images were used to test each calculation method. Of these data, 18 were abdomen scans and 17 were thorax scans. The selected scans had a localizer radiograph with no truncation of tissue. Sets of axial “non‐contrast” or “soft tissue” slices were used for attenuation analysis. Slices were mostly from full‐field‐of‐view reconstructions, with a few exceptions that had a small amount of skin truncation.

### 2.B Calculation and comparison of *D*_w_ values

Calculation of *D*_w_ values using the whole scan range and center‐slice attenuation methods, as well as the center‐slice geometry method, was carried out by scripts written in MATLAB (Natick, MA). *D*_w_ values from a localizer geometry method developed by Philips Healthcare (Andover, MA) were also compared.

**Figure 1:**
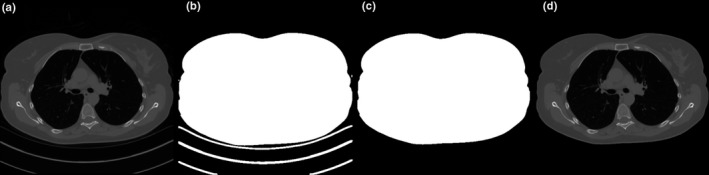
The isolation process. (a) The original image. (b) A mask is applied using the Sobel edge detection method. (c) Only the largest area is counted: this is the region‐of‐interest area used to calculate *D*_w_. (d) The mask and original image are combined: this image is used to calculate the average CT number of the body area.

Each method used involved the use of an edge detection image analysis algorithm to separate patient anatomy from background structures such as the table and padding. In each MATLAB script, Sobel edge detection was used, as shown in Fig. 1. The threshold for determining the pixel value difference that defined the outside of the patient was varied by data set and edge detection was inspected visually to confirm that it matched the visual border. The localizer analysis process was not available and proper edge detection could not be confirmed.

#### 2.B.1 Implementation of attenuation measurement methods

Each pixel in a reconstructed image contains information of the attenuation (attenuation coefficient *μ*) of x rays through the corresponding volume in the form of a CT number. AAPM TG220^4^ outlines a method for using these data to calculate *D*_w_, which is an ideal metric for estimating a patient's radiation absorption properties because it uses the patient's attenuation information directly.

CT numbers are defined relative to the attenuation coefficient of water, so they can be used to calculate the cross‐sectional area (*A*_*w*_) of a cylinder of water with average attenuation equivalent to that of the body in the analyzed slices [eqs. (1), (2), (3)].^4^

(1) $$A_{w}=\sum \left(\frac{\mu (x,y)}{\mu_{\mathrm{water}}}\right)\times A_{\mathrm{pixel}}$$

(2) $$=\sum \left(\frac{CT(x,y)}{1000}+1\right)\times A_{\mathrm{pixel}}$$

(3) $$=\left(\frac{\bar{CT(x,y)}}{1000}+1\right)\times A_{\mathrm{ROI}}$$

In these equations, $\mu_{\mathrm{water}}$ and μ(x,y) are the attenuation coefficients of water and of the tissue in the voxel denoted by the coordinates (*x*,* y*) of the slice, respectively. $A_{\mathrm{pixel}}$ is the area of one pixel, recorded in the DICOM data, and $A_{\mathrm{ROI}}$ is the area of the region of interest, determined by image analysis. CT(x,y) is the CT number of voxel (x,y), and $\bar{CT(x,y)}$ is the average CT number value of the slice.

The diameter of this area is *D*_w_ [eqs. (5), (6)].

(4) $$D_{w}=2\sqrt{\frac{A_{w}}{\pi }}$$

(5) $$=2\sqrt{\frac{A_{\mathrm{ROI}}}{\pi }\left(\frac{\bar{CT(x,y)}}{1000}+1\right)}$$

*D*_w_ is then related to a correction factor using tables from AAPM Task Group 220.^4^ The reported patient *D*_w_ is the average *D*_w_ of all slices of the desired analysis range.

##### Full scan range attenuation measurement

The reference method, to which the three other estimation methods were compared, used attenuation data from all slices along the full scan length. Using complete attenuation information from the whole scan provided the most accurate *D*_w_ value. The *D*_w_ of each slice was measured and recorded, then averaged to give the *D*_w_ value of the patient.

##### Central slice attenuation measurement

*D*_w_ estimation using analysis of few center slices was compared, with an analysis range of the scan's central 0.1 cm. This included between one and three slices, depending on reconstruction settings. Again, the *D*_w_ of each slice in the range was measured and averaged to give the *D*_w_ value of the patient.

#### 2.B.2 Implementation of slice geometry measurement method

The method implemented by the American College of Radiology's Dose Index Registry to obtain SSDE values for submitted scans is based on TG204's recommendation of using a combination of patient dimensions to calculate effective diameter.^10^ TG204 states that *D*_w_ can be calculated as a function of the sum of AP and LAT dimensions using multiplication factors determined by fitting data from multiple experiments and simulations.^3^

**Figure 2:**
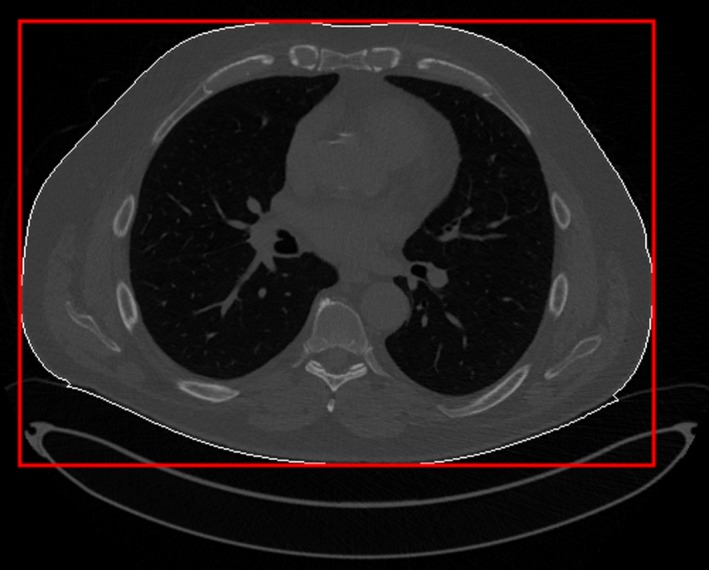
How patient dimensions are found using a central slice. The white line shows the border of the region found by edge detection. The red box is fit to this border; its dimensions are the AP and LAT measurements used to estimate *D*_w_.

A MATLAB script was written to locate the central slice and isolate the body area using the same edge detection as the attenuation method. LAT and AP dimensions were measured by taking the dimensions of the bounding box of the body area, as shown in Fig. 2. These dimensions were summed and input into eq. (6) to calculate *D*_w_.

(6) $$D_{w}=a+bx+cx^{2}$$

In this equation, *x* is the sum of the AP and LAT dimensions, and *a* = −0.203128, *b* = 0.4958912, *c* = 0 as determined by TG204.^3^

#### 2.B.3 Implementation of localizer radiograph geometry measurement method

The method of measuring patient geometry from the localizer radiograph begins by isolating the body region and removing any background structures using an edge detection algorithm. The lateral dimension of the patient was measured along the scan length and the *D*_w_ of these slices was calculated using interpolation between points in table from TG204,^3^ which relates the LAT dimension to a *D*_w_ value. These values were then averaged to obtain the final estimate of the patient's *D*_w_.

The processing of each image using this method was not observed in this study. Only the initial localizer image and the final *D*_w_ value were available.

### 2.C. Comparison of methods

Estimation method *D*_w_ values and the reference *D*_w_ obtained using the full scan attenuation data were compared directly using the mean difference (signed) and mean absolute difference (positive). The distribution of each set of differences was compared using a nonparametric two one‐sided test of equivalence (TOST) adopted from Mara et al.^11^ and carried out using Microsoft Excel. A TOST does not assume no difference as the null hypothesis, but rather presents the burden of proving equivalence. TOSTs can also indicate whether a method has a bias upward or downward compared to the reference method. The TOST provides left‐side and right‐side z‐scores, for which values greater than 2.58 correspond to significant results (*P *<* *0.01). Both sides must show significance to conclude equivalence within a specified margin.

Task Group 220 stated that *D*_w_ calculation using attenuation data from a localizer radiograph should be within 20% of the reference value.^4^ We use this 20% margin as the basis for comparison between estimated *D*_w_ values and the reference value, as well as for the equivalence margin of the two one‐sided test.

## 3 RESULTS

### 3.A Central slice attenuation method

**Figure 3:**
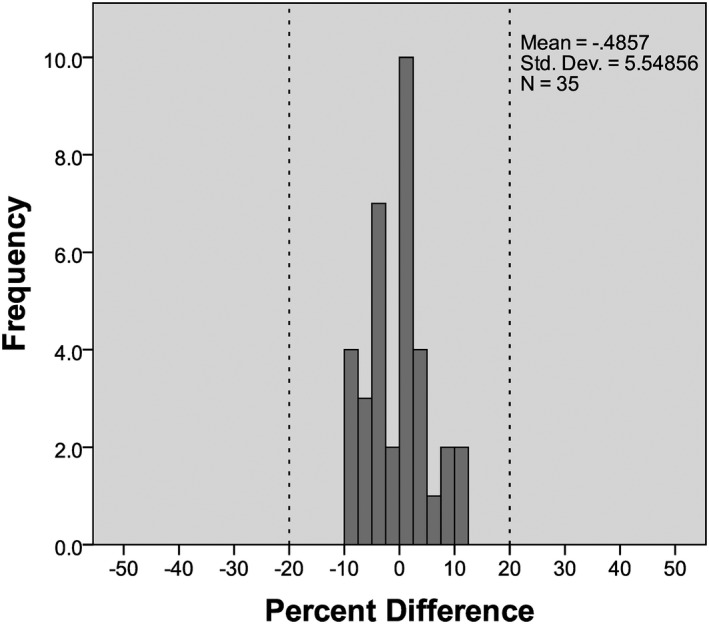
Histogram of percent differences between *D*_w_ values calculated using the central slice attenuation method and the reference method. A 20% margin is marked by dashed lines.

Using attenuation data from few reconstructed slices in the center of the scan range produced estimated *D*_w_ values with a mean difference from reference value of 0.13 ± 1.57 cm (mean absolute difference 1.26 ± 0.90 cm), with a range of differences between −2.59 and 3.38 cm. All *D*_w_ values calculated using attenuation data from a center slice are within 20% of their reference value, as demonstrated in Fig. 3. TOST analysis showed that the median values of the *D*_w_ distributions for this method and the reference method were equivalent within the test margin (*z*_right_ = 5.159, *z*_left_ = 5.159).

### 3.B Central slice geometry method

**Figure 4:**
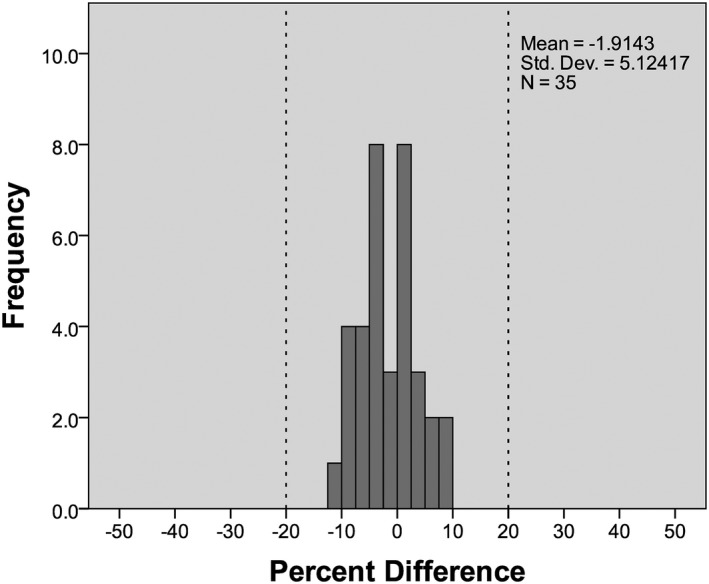
Histogram of percent differences between *D*_w_ values calculated using the center slice geometry method and the reference method. A 20% margin is marked by dashed lines.

A comparison of *D*_w_ values calculated using the reference method and those calculated using the sum of the AP and LAT dimensions of a central reconstructed slice showed a mean difference of −0.53 ± 1.50 cm (mean absolute difference 1.28 ± 0.90 cm), with a range of differences between −3.52 and 2.70 cm. As shown in Fig. 4, all *D*_w_ values calculated using the center slice geometry method are within 20% of their reference value. The TOST showed that the median values of the *D*_w_ distributions for this method and the reference method were equivalent within the test margin (*z*_right_ = 5.159, *z*_left_ = 5.159).

### 3.C Localizer radiograph method

**Figure 5:**
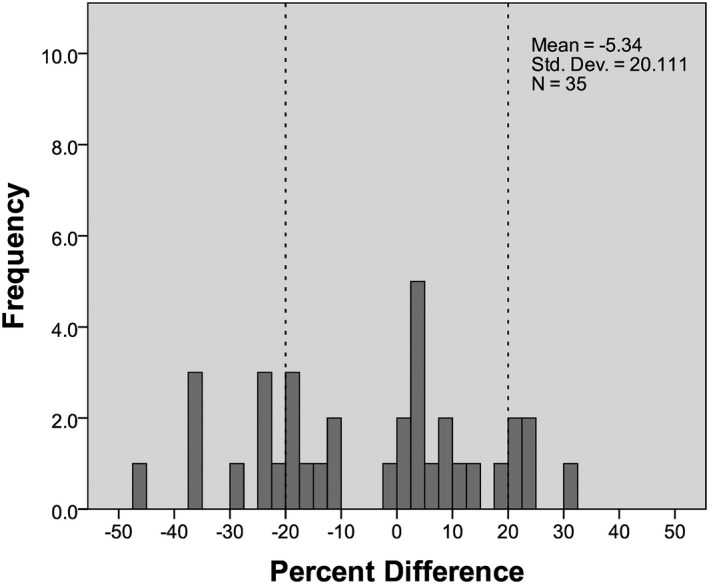
Histogram of percent differences between *D*_w_ values calculated using the localizer radiograph geometry method and the reference method. A 20% margin is marked by dashed lines.

A comparison between the reference D_w_ value (from full scan range attenuation analysis) and the D_w_ value estimated using localizer geometry showed a mean difference of −1.30 ± 5.72 cm (mean absolute difference 4.80 ± 3.28 cm). Differences ranged between −12.14 cm and 9.92 cm. Twenty‐two (62%) of the Dw values calculated with the localizer geometry method are within 20% of their reference value, as shown in Fig. 5. The TOST showed that the median values of the Dw distributions for this method and the reference method were equivalent within the test margin (*z*_right_ = 4.776, *z*_left_ = 3.734).

## 4 DISCUSSION

### 4.A Possible issues with *D*_w_ estimation methods

#### 4.A.1 Image edge detection

All methods implemented in this study used edge detection in their image analysis procedures. Difficulty arose when trying to include the whole body area while excluding external structures. Neighboring structures inside the body have different enough attenuation that analysis software can mistake the border between them as an external interface and exclude tissue from the region of interest. The same edge detection settings may not work across multiple images. Edge detection in localizer radiograph images is further complicated by the lack of absolute units in their grayscale values.^4^ Whereas CT numbers are related to the attenuation coefficient of water, grayscale values in localizer images are not standardized and may vary by machine, so setting a universal threshold for which values correspond to tissue is not feasible.

A disproportionate number of the *D*_w_ estimations outside the 20% margin (9 of 13, vs 17 of 35 in the whole study) are underestimates, which may suggest that the localizer radiograph implementation is prone to misinterpreting the edge of the patient region in the radiograph. Reducing the rate of tissue exclusion due to incorrect edge detection would greatly reduce the rate of major differences.

#### 4.A.2 Availability of complete attenuation information

In clinical situations, a physician may only require reconstruction of a small internal region. When a reconstruction of the patient along the whole scan range is not available, the method of calculating *D*_w_ from attenuation data will not yield an accurate result. Using the attenuation data from an incomplete reconstruction will produce a smaller *D*_w_, and overestimate the final SSDE value. A localizer radiograph image of the full scan area is always available, so a method that accurately estimates patient size using this image would not be subject to truncation issues.

Any *D*_w_ estimation method that averages data or uses a smaller data set to represent the whole is susceptible to inaccuracy when analyzing a patient with large variations in anatomical shape along the scan range. A method that measures one to three central slices is especially vulnerable to this error, so caution should be taken when dealing with such cases.

### 4.B Impact of measurement inaccuracy on SSDE

**Figure 6:**
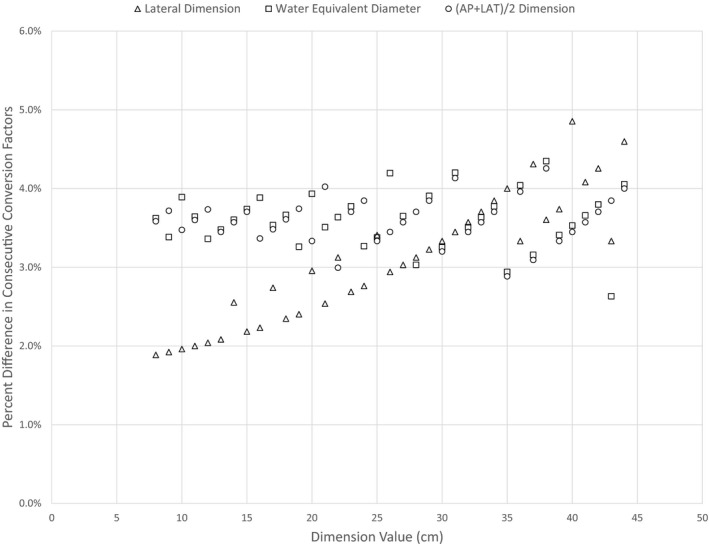
Percent difference between consecutive conversion factors *i* and (*i* + 1) as a function of the measurement dimension of the *i*th factor. Percent change for *D*_w_ and (AP + LAT)/2 dimensions stay mostly between 3 and 4% across all patient sizes, though spread increases. Using LAT measurement, the percent change increases with patient size.

Error in SSDE increases with the error of the measured value used to determine the CTDI_vol_‐to‐SSDE conversion factor. However, conversion factors given by tables in TG220 scale differently depending on the dimension that is considered. Figure 6 shows how the percent difference between consecutive conversion factors changes with the value of the measured dimension. These differences accumulate when measurement error is >1 cm.

Factors corresponding to the LAT dimension change uniformly by around 0.05 per centimeter, increasing the percent difference between consecutive factors as LAT increases (under 2% per centimeter for 8–9 cm, up to over 4% for 44–45 cm). Thus an inaccurate LAT measurement causes more error in SSDE calculation for a patient with a high LAT dimension vs a patient with a low LAT dimension. Another consequence is that underestimation of LAT causes less error per centimeter of inaccuracy than does overestimation.

When using *D*_w_ or AP + LAT values to determine the proper conversion factor, the change between consecutive factors decreases as measured value increases. The difference between consecutive conversion factors is a consistent 3–4% across all patient sizes, though spread increases. The error in SSDE per centimeter inaccuracy in the measured dimension is more uniform across patient sizes, but error estimation is less precise for larger patients. Below a dimension value of 25, measurement inaccuracy when using the LAT dimension alone has less impact than when using *D*_w_ or AP + LAT.

As an example, suppose a patient is determined to have a size dimension of 15 ± 2 cm, with uncertainty due to possible error in the measurement method. If that measurement is the LAT dimension, the patient would be given a size‐specific correction factor *k *=* *2.29 based on tables from AAPM TG 220.^4^ If the true value is 2 cm greater or smaller, this correction factor differs from the true factor by about 4.6%. If the measurement is *D*_w_, the correction factor is *k *=* *2.14 with a possible difference from the true factor of 7–8%, depending on whether the true size is smaller or greater. If the measurement is half of the AP + LAT dimension, *k *=* *2.16 with a possible difference from the true factor of 7–7.5%.

Compare this to a patient with a measured dimension of 30 ± 2 cm: the LAT‐based correction factor is *k *=* *1.5 with possible deviation of 6–7%. The *D*_w_‐based factor is 1.23 with possible deviation of 7–8%; and the AP + LAT‐based factor is 1.25 with possible deviation of 7.5–8%. The *D*_w_ and AP + LAT‐based correction factors for the larger patient have an uncertainty similar to the factors for the smaller patient. The uncertainty in the LAT‐based factor is comparable for the larger patient, but smaller for the smaller patient. Potential errors under 10% may not be worth considering in a clinical setting, but methods that can incorrectly measure patient dimensions by many centimeters introduce substantial error in dose estimation.

## 5 CONCLUSIONS

This study demonstrates that *D*_w_ estimation methods using the geometry or attenuation data from a central, full‐field‐of‐view reconstructed slice consistently produce results within 20% of their reference values and comply with the guidelines set by TG220. However, the localizer radiograph geometry method resulted in a considerable number of scans (13 of 35, 37%) that deviated by more than 20% of the reference value. The authors suggest that edge detection methods employed by localizer radiograph geometry methods should be fully evaluated prior to implementation in a clinical setting.

Although the method using reconstructed images are more accurate, full‐field‐of‐view reconstructions are not always available, and incomplete reconstructions can lead to SSDE overestimation. Localizer geometry‐based methods do not calculate *D*_w_ exactly, but a proper implementation could produce sufficiently accurate dose estimates using the localizer radiograph, which is already available, while avoiding outliers caused by varying reconstruction practices. This is especially true for smaller patients, since there is less variation in SSDE conversion factors at low values of the lateral dimension when it is the sole metric for patient size. When both AP and LAT localizer images are available, a localizer geometry analysis method could meet or exceed the accuracy of a central slice geometry method, which is already highly accurate for patients with uniform anatomy.

## CONFLICTS OF INTEREST

Andrew Daudelin was a paid intern of Philips Healthcare (Andover, MA), which provided data from their localizer radiograph analysis method, during this study. Chris Martel is an employee of Philips Healthcare.
